# Tuning the Degradation Rate of Alginate-Based Bioinks for Bioprinting Functional Cartilage Tissue

**DOI:** 10.3390/biomedicines10071621

**Published:** 2022-07-07

**Authors:** Xavier Barceló, Kian F. Eichholz, Orquidea Garcia, Daniel J. Kelly

**Affiliations:** 1 Trinity Centre for Biomedical Engineering, Trinity Biomedical Sciences Institute, Trinity College Dublin, D02 R590 Dublin, Ireland; barcelgx@tcd.ie (X.B.); eichholk@tcd.ie (K.F.E.); 2 Department of Mechanical, Manufacturing & Biomedical Engineering, School of Engineering, Trinity College Dublin, D02 R590 Dublin, Ireland; 3 Advanced Materials & Bioengineering Research Centre (AMBER), Royal College of Surgeons in Ireland & Trinity College Dublin, D02 F6N2 Dublin, Ireland; 4 Johnson & Johnson 3D Printing Innovation & Customer Solutions, Johnson & Johnson Services, Inc., Irvine, CA 92618, USA; ogarci22@its.jnj.com; 5 Department of Anatomy, Royal College of Surgeons in Ireland, D02 YN77 Dublin, Ireland

**Keywords:** bioprinting, bioink, oxidized alginate, degradation, tissue engineering, cartilage

## Abstract

Negative foreign body responses following the in vivo implantation of bioprinted implants motivate the development of novel bioinks which can rapidly degrade with the formation of functional tissue, whilst still maintaining desired shapes post-printing. Here, we investigated the oxidation of alginate as a means to modify the degradation rate of alginate-based bioinks for cartilage tissue engineering applications. Raw and partially oxidized alginate (OA) were combined at different ratios (Alginate:OA at 100:0; 75:25; 50:50; 25:75; 0:100) to provide finer control over the rate of bioink degradation. These alginate blends were then combined with a temporary viscosity modifier (gelatin) to produce a range of degradable bioinks with rheological properties suitable for extrusion bioprinting. The rate of degradation was found to be highly dependent on the OA content of the bioink. Despite this high mass loss, the initially printed geometry was maintained throughout a 4 week in vitro culture period for all bioink blends except the 0:100 group. All bioink blends also supported robust chondrogenic differentiation of mesenchymal stem/stromal cells (MSCs), resulting in the development of a hyaline-like tissue that was rich in type II collagen and negative for calcific deposits. Such tuneable inks offer numerous benefits to the field of 3D bioprinting, from providing space in a controllable manner for new extracellular matrix deposition, to alleviating concerns associated with a foreign body response to printed material inks in vivo.

## 1. Introduction

Tissue engineering aims to regenerate damaged or diseased tissues by combining supportive biomaterials, biochemical and/or physical signals and cells, often referred to as the tissue engineering triad [1,2]. Many tissue engineering strategies rely on the use of a biocompatible polymeric scaffold in which cells deposit the extracellular matrix (ECM) of interest. These polymeric matrices are expected to provide a conducive environment for the cells to proliferate and differentiate [3,4]. 3D bioprinting is an emerging biofabrication technique enabling the engineering of constructs mimicking the shape, spatial composition and organization of complex tissues [5,6,7,8,9]. Extrusion-based 3D bioprinting strategies have undergone extensive development in recent years and are now widely used for producing 3D tissue constructs [8]. To enable this, numerous different hydrogel inks have been developed, including synthetic (e.g., poly(ethylene glycol) and pluronic) and natural polymers (e.g., alginate and agarose), protein-based (e.g., collagen and fibrinogen) and native tissue-derived materials (e.g., tissue-specific extracellular matrix) [10,11,12,13,14]. A common goal when designing biomaterials for tissue engineering applications is that they are gradually replaced with newly deposited ECM, without detrimentally affecting the structural stability of the overall construct [15,16]. In the field of cartilage tissue engineering, it is known that in vitro and in vivo tissue development is strongly dependent on the rate at which the supporting hydrogel degrades [17,18,19]. However, little is known about how the degradation kinetics of bioinks will impact the development of bioprinted cartilaginous tissues, or how material modifications introduced to alter the rate of degradation might impact bioink rheology and hence their printability.

**Figure 1:**
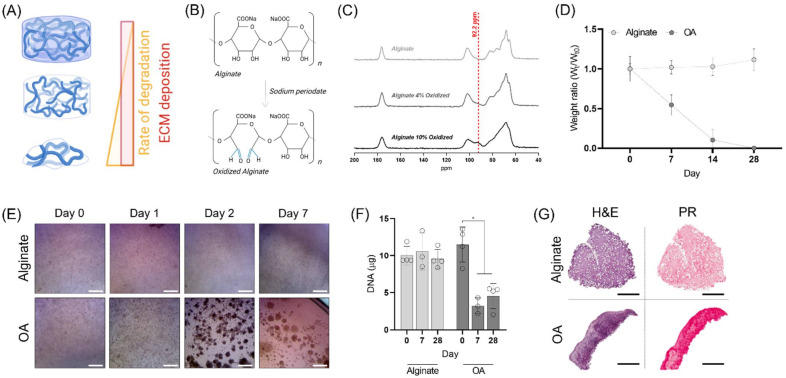
Oxidized alginate hydrogels. (**A**) ECM deposition in hydrogels presenting different degradation rates. (**B**) Reaction scheme of the alginate oxidation using sodium periodate. (**C**) 13C NMR spectra for alginates with different degradation rates. (**D**) Dry weight of oxidized alginate and raw alginate at various time points. (**E**) Macroscopic views of the cell-laden hydrogels at different time points. The scale bars are equal to 500 μm. (**F**) DNA quantification in each construct at Day 0, Day 7, and Day 28. (**G**) Hematoxylin and eosin (H & E) and picrosirius red (PR) stainings after 28 days in culture. The scale bars are equal to 1 mm. * indicates significance *p* < 0.05.

Alginate is a natural polymer commonly used in the 3D bioprinting of engineered cartilaginous tissues [7,20,21,22]. It undergoes a relatively rapid and gentle gelation through the interaction with divalent cations, which makes it attractive for cell encapsulation [23]. A potential limitation of alginate for certain bioprinting and tissue engineering applications is that mammalian cells do not produce enzymes capable of degrading the polymer, leading to a slow degradation process that is primarily controlled by the outward flux of ions into the surrounding environment. In orthopaedic applications, it has been shown that large amounts of residual alginate are still present 8 weeks after in vivo implantation, demonstrating the slow rate at which this material degrades [24]. A number of strategies have been investigated to develop alginate-based hydrogels with more controlled degradation kinetics, such as the use of gamma irradiation, oxidation, lower polymer densities, or the use of calcium chelating compounds such as sodium citrate to induce the dissolution of the hydrogel [25,26,27,28]. Although such approaches can be leveraged to accelerate the degradation of the hydrogel, balancing the rate of biomaterial degradation with the rate of newly deposited ECM is challenging and tissue-specific. In the context of developing an alginate-based biomaterial for 3D bioprinting of cartilaginous tissues, an ink should not only be cell-compatible and printable, but should also degrade at an appropriate rate. The degradation should be fast enough to provide space for cells to deposit abundant new ECM, however, it should not be excessively fast such that the printed construct will lose its mechanical integrity and initial structure (Figure 1A).

In the present study we investigated the development of alginate-based bioinks with different degradation rates by combining networks of partially oxidized and raw alginates capable of retaining the 3D printed structure while simultaneously providing an environment supportive of rapid cartilage tissue development. We also explored how the inclusion of temporary viscosity modifiers (specifically gelatin) would influence the printability of such alginate-based inks. After characterizing the degradability, mechanical properties and biocompatibility of various bioink formulations, we then assessed their relative capacity to support chondrogenesis of mesenchymal stem/stromal cells (MSCs) and the development of functional cartilage tissues in vitro.

## 2. Materials and Methods

### 2.1. Oxidized Alginate (OA) Synthesis

OA was prepared as previously described [29]. Briefly, 1 g of MVG sodium alginate (Pronova Biopolymers, Halland, Sweden) was dissolved in 90 mL of ultrapure deionized water overnight at 37 °C, and mixed with 10 mL of sodium periodate (Honeywell, Charlotte, NC, USA) at different concentrations to achieve different degrees of theoretical alginate oxidation (Table 1). The solution was continuously stirred in the dark at room temperature (RT) for 24 h, followed by a dialysis step against deionized water for 3 days (MWCO 3500 Dalton; Fischer, Waltham, MA, USA). The solution was then sterile-filtered through 0.22 µm filter, after which it was lyophilized. The increased degree of oxidation was assessed through NMR spectroscopy (Bruker Avance II, MA, USA) at RT.

### 2.2. Isolation and Expansion of Mesenchymal Stem/Stromal Cells

Bone marrow derived MSCs were obtained from the femur of a 4-month old pig as previously reported [30]. Briefly, under sterile conditions, the bone marrow was removed from the femoral shaft, and mixed with expansion medium (XPAN), consisting of hgDMEM supplemented with 10% *v/v* FBS, 100 U/mL penicillin, 100 µg/mL streptomycin, and 2.5 µg/mL amphotericin B (all Gibco, Biosciences, Dublin, Ireland). A homogenous suspension was achieved by triturating with a 16G needle. The suspension was then filtered through a 40 μm nylon mesh. Cells were counted and seeded into dishes for colony forming assay, or into T175 flasks for expansion. All expansion was conducted in hypoxic conditions at 5% O_2_, and using XPAN medium containing 5 ng/mL FGF2 (Peprotech, London, UK) [31]. The tripotentiality of isolated MSCs was assessed using chondrogenic, osteogenic and adipogenic differentiation assays. Cells of passage 3 were used for experiments.

### 2.3. 3D Bioprinting Process

Cell-laden bioinks were prepared by mixing a 5.3% (*w/v*) alginate solution and a 21.5% (*w/v*) gelatin solution (Gelatin type B; Sigma-Aldrich, Wicklow, Ireland) with a cell suspension (everything in hgDMEM) to obtain a final solution of 3.5% (*w/v*) of alginate and 5% (*w/v*) of gelatin containing 20 × 10^6^ cells/mL. For acellular characterisation, the volume of cell suspension was substituted for hgDMEM to maintain the same polymer concentrations. The different bioinks were loaded into a syringe, and then printed using the 3D Discovery bioprinter (RegenHU, Villaz-St-Pierre, Switzerland) at 16 °C, using a pressure of 0.125 MPa, a translational speed of 4 mm/s and a plastic conical needle of 25G (Adhesive Dispensing Ltd., Buckinghamshire, UK). Cellular constructs were printed following a cylindrical geometry of 4 mm in diameter by 4 mm height, following a grid pattern with strand distance of 0.250 mm and a z distance increase of 0.250 mm. After printing, the different samples were incubated for 30 min in a bath of 45 mM CaCl_2_ in hgDMEM, and then the cell-laden hydrogel were maintained in XPAN medium for 24 h before switching to chondrogenic medium (CDM) (Table 2) at 5% O_2_. Media exchange was performed twice weekly until the end of the 4-week culture period.

### 2.4. Live/Dead Imaging

Cell viability was assessed via a live/dead assay at day 7 after bioprinting. Briefly, constructs were washed in phenol-free DMEM (pfDMEM; Sigma-Aldrich, Wicklow, Ireland) followed by incubation in pfDMEM containing 2 μM calcein acetoxymethyl (calcein AM) and 4 μM ethidium homodimer-1 (EthD-1) (both from Bioscience, Dublin, Ireland) for 1 h. Samples were washed in pfDMEM before imaging with a Leica SP8 scanning confocal microscope (Wetzlar Germany) excited at 494 nm and 528 nm, and read at 517 nm and 617 nm.

### 2.5. Biochemical Analysis

The biochemical content of the constructs were analysed after 4 weeks of in vitro culture. Constructs were washed in deionized water, frozen, and freeze-dried to obtain the dry weight. Bioprinted constructs were digested using a 3.88 U/mL papain solution in ultra-pure water containing 5 mM L-cysteine–hydrochloride hydrate, 0.1 M sodium acetate, 5 mM methylenediaminetetraacetic acid (EDTA) (all from Sigma–Aldrich, Wicklow, Ireland). The pH was adjusted to 6.5 using 38% HCl. Samples were incubated in papain solution at 60 °C, rotating at 10 rpm overnight. DNA content was quantified immediately after digestion using the DNA Quantification Kit (Merck, Dublin, Ireland). The amount of sulphated glycosaminoglycan (sGAG) was determined using the dimethylmethylene blue dye-binding assay, with a chondroitin sulfate solution (Blyscan, Biocolor Ltd., Carrickfergus, UK) for the standards. The pH of the DMMB was adjusted to 1.5 using a 12 N HCl solution. Total collagen content was quantified by determining the hydroxyproline content of the constructs using the dimethylaminobenzaldehyde and chloramine T assay, and calculated assuming a hydroxyproline-to-collagen ratio of 1:7.69 [32].

### 2.6. Histological Analysis

Constructs were fixed in 4% paraformaldehyde, dehydrated in a graded series of ethanol and xylene baths, embedded in paraffin wax, sectioned at 8 μm using a microtome (Leica Microsystems, Wetzlar Germany), and affixed to microscope slides. The sections were stained with hematoxylin and eosin (H&E), alcian blue, picrosirius red and alizarin red. Antigen retrieval was carried out by an initial treatment with pronase (3.5 U/mL; Merck, Dublin, Ireland) at 37 °C for 25 min, followed by hyaluronidase (4000 units/mL; Sigma-Aldrich, Wicklow, Ireland) at 37 °C for 25 min for collagen type I and type II. For collagen type X, the antigen retrieval method consisted of an initial treatment with pronase (35 U/mL; Merck, Dublin, Ireland) at 37 °C for 5 min, followed by chondroitinase ABC (0.25 U/mL; Sigma-Aldrich, Wicklow, Ireland) at 37 °C for 45 min. Non-specific sites were blocked using a 10% goat serum and 1% BSA blocking buffer for 1 h at room temperature. Collagen type I (1:400; ab138492; Abcam, Cambridge, UK), type II (1:400; sc52658; Santa Cruz, Heidelberg, Germany), and type X (1:300; ab49945; Abcam, Cambridge, UK) primary antibodies were incubated ON at 4 °C, followed by 20 min treatment using a solution of 3% hydrogen peroxide (Sigma-Aldrich, Wicklow, Ireland). The secondary antibodies for collagen type I (1:250; ab6720; Abcam, Cambridge, UK), type II (1:300; B7151; Sigma-Aldrich, Wicklow, Ireland) and type X (1:500, ab97228; Abcam, Cambridge, UK) were incubated for 4 h at RT. Samples were then incubated for 45 min with VECTASTAIN Elite ABC before treating them with ImmPACT DAB EqV (both from Vector Labs, Newark, NJ, USA) at RT. Slides were then imaged using an Aperio ScanScope slide scanner.

### 2.7. Scanning Electron Microscopy (SEM)

For SEM imaging, samples were frozen in liquid nitrogen, and immediately dehydrated by lyophilisation at −10 °C overnight. Samples were cut in half and mounted on SEM pin stubs with carbon adhesive discs and coated with gold/palladium for 60 s at a current of 40 mA using a Cressington 208HR sputter coater. Imaging was carried out in a Zeiss ULTRA plus SEM.

### 2.8. Rheological Assessment of Bioinks 

The rheological properties of all the bioinks were evaluated using a rheometer (MCR 102, Anton-Paar, Dublin, Ireland) equipped with a peltier element for temperature control. A plate-plate geometry with a diameter of 25 mm (PP25) was used in all the tests. The viscosity as a function of shear rate (0.1 to 1000/s) was conducted at a constant temperature of 16 °C. Then, the gelation kinetics were assessed with a temperature sweep from 37 °C to 4 °C with an increment rate of 5 °C/min while maintaining the shear rate at 1/s. Bioinks were kept in a high humidity atmosphere to prevent dehydration from affecting the rheological results.

### 2.9. Degradation Rates of Alginates and Gelatin Elimination Analysis

To determine the degradation rate of the different bioinks cylindrical-shaped constructs of 5 mm diameter and 3 mm height were printed as described above. After crosslinking with CaCl_2_, the constructs were then transferred into a 24-well plate, and cultured for 28 days at 37 °C. Media was changed twice every week using hgDMEM. At different time points, samples were washed with deionized water and frozen at −80 °C. The samples were lyophilized to obtain the dry weight. To investigate the presence of gelatin after the incubation of the hydrogels at 37 °C, a hydroxyproline assay was performed as described in the biochemical analysis methods.

### 2.10. Mechanical Testing 

To investigate how the different degrees of oxidation and blends influence the mechanical properties of the final gel, constructs were 3D printed as described previously. Mechanical compression tests were performed using a single column Zwick (Zwick, Rowell, Hinsdale, IL, USA) with a 10 N load cell in wet conditions using a PBS bath as previously reported [33]. The Young’s modulus was defined as the slope of the linear phase of the resulting stress-strain curve during the ramp phase of the compression to 10% strain.

### 2.11. Statistical Analysis

GraphPad Prism software (San Diego, CA, USA) was used to perform all the statistical analysis. Results are displayed as mean ± standard deviation, and were analysed by analysis of variance (ANOVA) followed by Tukey’s multiple comparison test or Student’s *t*-test Significance was determined at *p* < 0.05. Sample size number (n) is specified within the respective figure legends.

## 3. Results

### 3.1. Alginate Oxidation and Characterization

Partial oxidation of alginate was carried out using sodium periodate (NaIO_4_) as an oxidizing agent. This chemical reaction leads to the formation of two aldehyde groups, and the cleavage of a carbon-carbon bond, creating an alginate that is hydrolytically labile (Figure 1B). By controlling the stoichiometric addition of NaIO_4_, with respect to the alginate content, the sugar monomers in the alginate chains were partially oxidized to different extents (1, 4, 5 and 10%). The 13C NMR spectra of different oxidized alginates displayed an increased peak at 92.2 ppm corresponding to the aldehyde groups (Figure 1C) [34]. The degradation profile of the different alginates was examined by maintaining alginate gels in standard cell culture conditions for 28 days (Figure 1D; Supplementary Figure S1A). The dry weight of the unmodified alginate and the 1% partially oxidized gels did not show any mass loss during the 4 weeks in culture, whereas the alginates with a 4% and 5% degree of oxidation had completely degraded after 28 and 14 days, respectively. We were unable to form hydrogels with the 10% oxidized alginates, and thus this condition was not further characterised. The compressive properties of the alginate-based hydrogels decreased as a function of increasing percentage of oxidation (Supplementary Figure S1B). The elastic modulus reduced from 29.03 ± 1.61 kPa for the unmodified alginate to 9.15 ± 3.60 and 3.68 ± 0.39 kPa for partial oxidation of 4 and 5%, respectively. As the 4% alginate underwent complete degradation over 4 weeks, a time period within the range typically used for in vitro cartilage tissue engineering studies, this condition was selected for further analysis and development. Such a degradation timeframe would also ensure that the majority of alginate material was cleared from a bioprinted tissue before its implantation into the body. From this point onwards, we will refer to the 4% oxidized alginate as ‘OA’.

We next evaluated the chondrogenic capacity of porcine MSCs encapsulated within OA and raw alginate to discard any detrimental effect due to the presence of aldehyde groups in the polymer backbone. Immediately after hydrogel encapsulation, no major differences in cell behaviour could be observed, with rounded cells observed in both conditions (Figure 1E). After 24 h, small clusters of cells started forming in the OA gels, which increased in size after 48 h and remained for the entire period of culture. The DNA content within the OA decreased during the first week of culture, and then remained constant throughout the 28-day culture period. Conversely, the DNA levels in unmodified alginate hydrogels did not change over time. Histological evaluation revealed that cells were distributed throughout the depth of the unmodified alginate hydrogels but appeared more clustered within the OA hydrogels (Figure 1G). The strong staining for new tissue deposition within both groups indicated that the presence of aldehyde groups did not affect the chondro-permissiveness of the gels (Figure 1G), with robust sGAG and collagen deposition observed in both groups (Supplementary Figure S2). However, the OA gels had lost their mechanical stability by the end of the culture period, suggesting that their rate of degradation was still too fast for the cell secreted ECM to stabilize the 3D structure.

### 3.2. Bioink Characterization

**Figure 2:**
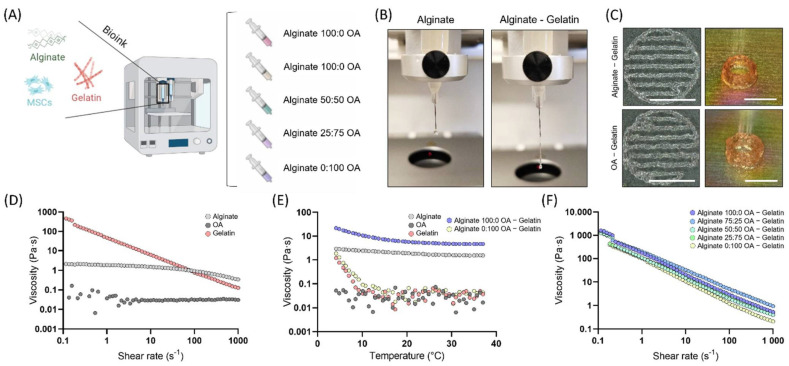
Bioink characterization. (**A**) Schematic of the experimental plan. Illustration of the bioink composition and different blends investigated. (**B**) Droplet/Filament formation demonstrating the role of gelatin for printing low viscous inks. (**C**) Printed hydrogels following grid and tubular patterns. Scale bar is equal to 6 mm. (**D**) Viscosity of the individual components (polymers) investigated under a shear rate in the range of 0.1 to 1000 s^−1^ at 16 °C. (**E**) Viscosity of bioinks and individual components as a function of temperatures changing from 37 to 4 °C. (**F**) Viscosity of the different bioinks in the presence of shear rate in the range of 0.1 to 1000 s^−1^ at 16 °C.

Having demonstrated the capacity of OA hydrogels to completely dissolve after 4 weeks, we sought to investigate the potential of combining slow (raw alginate) and fast (OA) degrading networks to obtain a more balanced degradation rate suitable for in vitro tissue engineering and 3D bioprinting (Figure 2A). For the purpose of this study 5 different hydrogel inks were designed by changing the ratio of raw alginate to OA (Alginate:OA): 100:0; 75:25; 50:50; 25:75; 0:100. The oxidation process greatly reduced the polymer viscosity compared to raw alginate, making it unsuitable for extrusion bioprinting (Figure 2D). In order to make the alginate-based inks printable, we incorporated gelatin as a temporary viscosity enhancer to enhance their rheological performance, which supported the development of a continuous filament during extrusion-based bioprinting (Figure 2B). This can be attributed to the higher viscosity of the gelatin, which displays a clear shear-thinning behaviour (Figure 2D). Due to the thermal-gelation properties, the viscosity of gelatin containing inks increased when the temperature was decreased from 37 to 4 °C, indicating that temperature can be used to tune the rheological properties of the bioinks (Figure 2E). After gelatin gelation at 16 °C, all the bioinks displayed similar rheological properties with a pronounced shear thinning behaviour (Figure 2F). For that reason, 16 °C was selected as the optimal temperature to carry out all bioprinting steps. The different gelatin containing bioinks could be successfully printed into patterns or tubular structures (6 cm Ø and 3 cm height) with a spreading ratio of 1.79 ± 0.17 for the alginate only bioink (100:0), and 1.93 ± 0.18 for the OA only bioink (0:100), see Figure 2C and Supplementary Figure S3.

### 3.3. Degradation Rate of the Blended Bioinks

**Figure 3:**
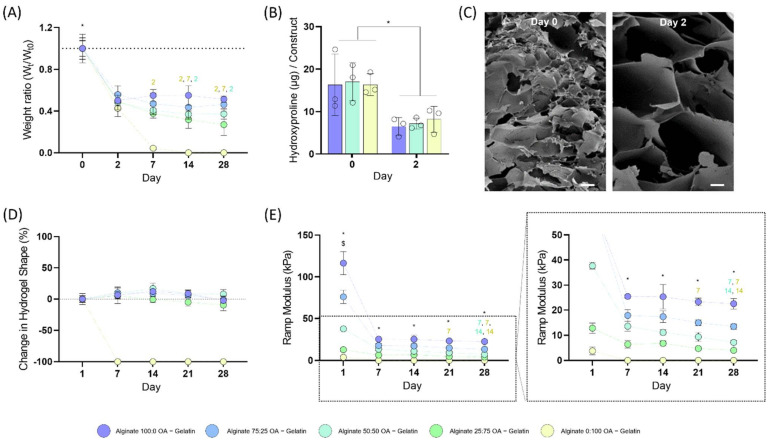
Hydrogel characterization. (**A**) Dry weight of 3D printed hydrogels at various time points. * different to all time points; 2,7 different to day 2 and day 7 (color equals condition). (**B**) Quantification of the hydroxyproline levels in each construct at day 0 and day 2. (**C**) SEM images at Day 0 and Day 2 revealing the increased porosity after the gelatin removal. The scale bars are equal to 20 μm. (**D**) Hydrogel shape tracking as a function of time. (**E**) Mechanical properties of the different bioinks at different time points. * all groups are different; 7, 14 different to day 7 and day 14 (color equals condition). $ different to all time points.

In order to assess the degradation profile of the different bioinks, cylindrical constructs were printed and maintained in culture for a 28-day period. A rapid decrease in dry weight was seen in all the bioinks after 48 h, which can be related to the release of gelatin after the incubation of the constructs at 37 °C (Figure 3A). As seen from the decrease in hydroxyproline content in each hydrogel, and the SEM images, most of the gelatin was released after 48 h, leaving behind pores in the alginate gels (Figure 3B,C; Supplementary Figure S4). At longer incubation times, the 100:0 and 75:25 gels displayed minimal additional mass loss. In contrast, the 50:50 and 25:75 gels displayed time-dependent reductions in their mass. Specifically, the 25:75 group lost a further 45% of its dry weight between day 2 and day 28, whereas the 50:50 had lost a further 23% of its mass within the same period of time. As expected, the only group which underwent a complete mass loss during this time period was the 0:100 group.

In terms of their mechanical stability, all the bioinks containing unmodified alginate were able to retain their initial cylindrical shape (measuring the base diameter and height of the construct; Figure 3D). We also mechanically characterised the different constructs across the 4-week time period (Figure 3E). The initial compressive modulus dramatically decreased in all bioink groups after 7 days. Statistically significant differences in stiffness were observed between the different groups at all time points, with stiffness decreasing as the amount of unmodified alginate was reduced. There was no significance decrease with time in the mechanical behaviour of the 100:0 constructs, which was consistent with the mass loss data. In contrast, the ramp modulus of the 75:25, 50:50 and 25:75 constructs decreased by 25, 47 and 37% over the 28-day period.

### 3.4. Cell Viability and Chondrogenic Differentiation

**Figure 4:**
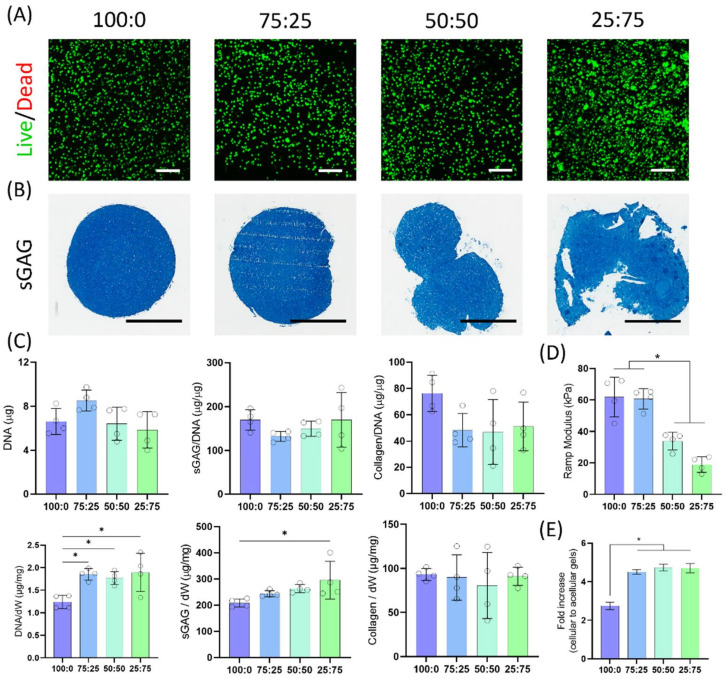
Chondrogenic evaluation of the bioinks. (**A**) Representative images of the live/dead staining at day 7. Green and red indicate live and dead cells, respectively. Scale bars are equal to 200 μm. (**B**) Alcian blue staining for sulphated glycosaminoglycan (sGAG). Scale bars are equal to 2 mm. (**C**) Quantification of DNA, sGAG and collagen deposition in each construct at day 28. (**D**) Ramp modulus of the constructs derived from the linear region of compressive strain-stress curves after 28 days in culture. (**E**) Fold change in the ramp modulus of acellular and cellular hydrogels following 4 weeks of in vitro culture. * indicates significance *p* < 0.05.

We next sought to evaluate the capacity of these different bioink blends to support chondrogenesis of encapsulated MSCs over 4 weeks in culture post-bioprinting. Live/dead staining after 7 days in culture revealed a highly viable cell population (Figure 4A, Supplementary Figure S5). Since the 0:100 construct had completely lost its shape and degraded within 7 days, no additional cell characterisation was performed on this group. As expected, cells displayed a round morphology within the alginate-based bioinks. In the 25:75 group, MSCs started forming small clusters as the hydrogel degraded. After 4 weeks of culture, alcian blue staining demonstrated high levels of sGAG deposition in all bioinks (Figure 4B). No differences in DNA content were seen across the groups (Figure 4C). Similarly, no differences in sGAG and collagen synthesis, when normalized to DNA, were observed across the groups. However, when normalised to the construct dry weight, significantly higher levels of sGAG were observed in the 25:75 group compared to the unmodified alginate bioinks.

Mechanical testing at the end of the 28-day culture period revealed that the stiffness was lower in OA containing constructs (Figure 4D). Compared to the acellular hydrogels at the same time point, the ramp modulus had increased 2.75 and 4.51 times for the 100:0 and 75:25, and 4.73 and 4.70 times for the 50:50 and 25:75, respectively (Figure 4E).

**Figure 5:**
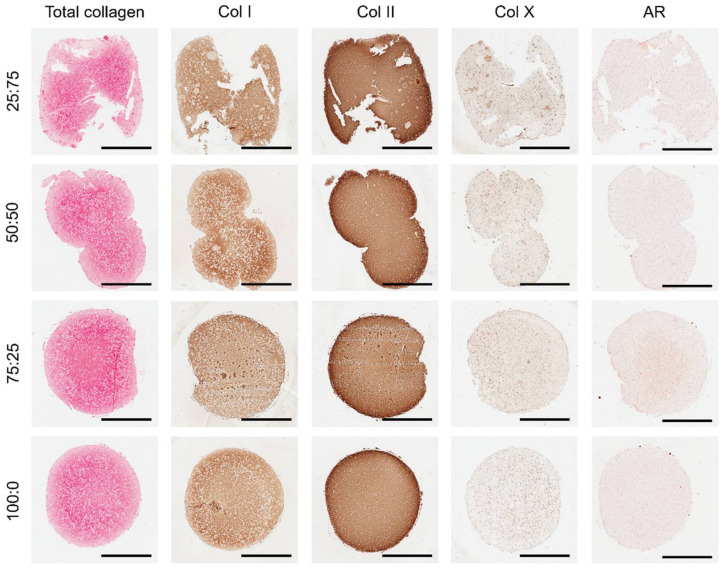
Histological evaluation. Picrosirius red staining for total collagen. Immunohistochemical staining for collagen types I, II, and X. Alizaring red (AR) staining for calcium deposits. Scale bars are equal to 2 mm.

Collagen staining further indicated robust chondrogenic differentiation of the encapsulated MSCs in all bioinks, with no calcium deposits observed as evident by negative staining for alizarin red (Figure 5). Immunohistochemical analysis revealed strong staining for collagen type II, with some type I deposition. Although negligible staining for collagen type X was observed in the 100:0, 75:25 and 50:50 groups after 4 weeks of culture, small pockets of tissue staining positive for this hypertrophic marker was observed in the 25:75 constructs, which correlated with the formation of cellular clusters.

## 4. Discussion

In this study, we sought to develop bioinks with temporally defined rates of degradation to support the in vitro formation of functional cartilaginous tissues. The combination of partially oxidized and unmodified alginate enabled finer control of hydrogel degradation, while maintaining the 3D structure of the printed construct in culture. The bioinks with higher OA contents were softer and underwent faster degradation, resulting in higher levels of cellular clustering. All of the alginate-based bioinks were found to support robust chondrogenesis of MSCs, with no major differences in cellular phenotype observed with changes in the rate of ink degradation. Taken together, these findings support the use of alginate-based bioinks with temporally defined rates of degradation to support the in vitro biofabrication of tissues such as articular cartilage, hypertrophic cartilage or meniscus. Designing inks that rapidly degrade upon the establishment of a functional repaired tissue will be integral to the development of successful bioprinting strategies, as it will help to mitigate concerns associated with a negative in vivo response (e.g., a foreign body response) to implanted materials.

As expected, the oxidation of the alginate polymer resulted in hydrogels with lower mechanical properties and faster degradation [35,36,37]. Partial oxidation of alginate using sodium periodate leads to the formation of aldehyde groups in the oxidized monomers, but also reduces the molecular weight of the polymer. Here, partial oxidation above 5% yielded OAs which could not form stable gels (e.g., 10% oxidation), whereas 5% oxidation rates resulted in weak gels that fully dissolved after 1 week (Supplementary Figure S1), which is consistent with previous studies [36]. For this reason, 4% oxidation was selected for further development since it would completely degrade relatively quickly (2–4 weeks), providing encapsulated cells with the space to condensate and secrete cartilaginous ECM components. However, the rapid degradation and low mechanical properties of the 4% oxidized alginate led to their physical collapse during in vitro culture, causing changes to the initial shape of the constructs and a loss of encapsulated cells (Figure 1E–G). This is a problem for 3D bioprinting strategies where maintaining a specific shape is integral to success.

In order to more tightly control the rate of ink degradation, we explored the blending of oxidized and non-oxidized alginates to obtain bioinks with different degradation rates. In many previous studies using alginate as a bioink, the use of a temporary viscosity enhancer such as gelatin or methylcellulose, or the addition of CaCl_2_ to partially crosslink the alginate pre-printing, is generally required to enable printing [22,28,38,39,40,41]. The incorporation of 5% (*w/v*) gelatin into the alginate blends conferred the bioinks with rheological properties compatible with extrusion bioprinting [42]. All gelatin containing bioinks displayed comparable rheological properties that permitted the use of similar 3D bioprinting methodologies and parameters, despite the fact that the alginate blends showed very different viscosities in isolation. After placing the constructs in the incubator at 37 °C (which is above the melt temperature for gelatin), the gelatin was gradually released from the hydrogel inks (Figure 3A,B). By measuring the dry weight and analysing the hydroxyproline content in the gels, we could determine that most of the gelatin was washed out after 2 days, leaving behind open pores in the alginate gels similar toobservations in previous works (Figure 3C) [28]. In spite of the gelatin release, the initial 3D printed geometry was maintained throughout the culture period for all constructs containing a fraction of unmodified alginate.

All the different bioinks supported high levels of cell viability and robust chondrogenic differentiation of MSCs. The different alginate blends supported comparable levels of sGAG and collagen deposition, demonstrating that the altered biophysical properties of the OA containing inks did not negatively impact their capacity to support robust new tissue formation. The amount of DNA and sGAG (per dry weight of the construct) was higher in the 25:75 group, which can be attributed to the fact the oxidized polymer is more susceptible to hydrolytic scission, thereby decreasing the hydrogel dry weight. The high levels of collagen type II seen across all groups confirms the development of a cartilage tissue, which can be linked to the absence of cell-binding motifs forcing cells to acquire a rounded morphology associated with the hyaline cartilage phenotype [7]. Interestingly, collagen type X staining was only detected in the group with larger amounts of OA where cells were allowed to cluster together. The formation of cell clusters is a hallmark of osteoarthritic cartilage formed after cell migration and/or proliferation, which express high levels of collagen type X [43]. The clustering of cells in these OA hydrogels can be linked to the rapid degradation of the gel, which facilitates cell migration and clustering. Hydrogel stiffness is another key factor which has been shown to regulate MSC chondrogenesis and hypertrophy. While previous studies have generally reported that stiffer hydrogel environments generally enhance the expression of markers associated with chondrocyte hypertrophy [44,45], it should be recognised that softer hydrogels that facilitate cell-cell interactions will generally accelerate chondrogenesis of MSCs and lead to associated increases in the expression of hypertrophic markers such as type X collagen [46].

The main limitation of this study is the fact that stiffness and degradation are coupled, which means faster degrading hydrogels are softer, increasing the number of variables that are known to influence cellular phenotype within such hydrogel systems. A possible solution to uncouple both degradation and stiffness is the use of binary alginate gels [35,47,48]. Using such systems, it is possible to generate hydrogels with comparable stiffness but presenting different degradation rates by combining alginates with different molecular weights and extents of oxidation. While not measured here, it is also likely that the viscoelastic properties of the bioinks (e.g., stress relaxation) also change with the ratio of unmodified alginate to OA, a factor which is also known to regulate chondrogenesis in vitro [49]. Future studies will look into developing a library of alginate-based bioinks with different degradation rates whilst also controlling for other key factors such as hydrogel stiffness and stress relaxation.

In summary, a strategy was developed to obtain bioinks that can act as temporal support structures for tissue engineering and bioprinting applications. Here, the partial oxidation of the alginate (4%) permitted its complete degradation after 4 weeks in culture. However, this rapid degradation led to a complete loss of the initial 3D structure, which was overcome by combining oxidized and unmodified alginates. The addition of 5% (*w/v*) gelatin to the alginate-based bioinks greatly improved their rheological behaviour, making them suitable for extrusion bioprinting. These tuneable bioinks were capable of structurally supporting the chondrogenic differentiation of MSCs for a period of 28 days without collapse or significant changes in the printed shape. In the context of tissue engineering and bioprinting, these temporal bioinks show significant potential for the in vitro biofabrication of tissues that are rapidly cleared of supporting exogenous biomaterials, ultimately resulting in ‘scaffold-free’ bioprinted tissues which can be more easily integrated in vivo.

## Figures and Tables

**Table 1:** Theoretical calculations for alginate oxidation.

Name	Theoretical Oxidation (%) *	Amount of NaIO_4_ (g)
Alginate	0	0
OA 1%	1	0.0108
OA 4%	4	0.0432
OA 5%	5	0.054
OA 10%	10	0.108

* Calculations were based on that the molecular weight of the alginate repeating unit is 198 g/mol.

**Table 2:** Composition of the chondrogenic medium.

Chondrogenic Medium (In hgDMEM)	
100 U/mL Penicillin (Gibco, Biosciences, Dublin, Ireland)	100 μg/mL Streptomycin (Gibco, Biosciences, Dublin, Ireland)
100 μg/mL Sodium pyruvate (Sigma-Aldrich, Wicklow, Ireland)	40 μg/mL L-proline (Sigma-Aldrich, Wicklow, Ireland)
50 μg/mL L-ascorbic acid-2-phosphate (Sigma-Aldrich, Wicklow, Ireland)	4.7 μg/mL Linoleic acid (Sigma-Aldrich, Wicklow, Ireland)
1.5 mg/mL Bovine serum albumin (BSA; Sigma-Aldrich, Wicklow, Ireland)	1 X Insulin–Transferrin–Selenium (Sigma-Aldrich, Wicklow, Ireland)
100 nM Dexamethasone (Sigma-Aldrich, Wicklow, Ireland)	2.5 μg/mL Amphotericin B (Sigma-Aldrich, Wicklow, Ireland)
10 ng/mL of human transforming growth factor-β3 (TGF-β3; Peprotech, London, UK)	

## Data Availability

The data presented in this study are available on request from the corresponding author.

## Supplementary Materials

The following supporting information can be downloaded at: https://www.mdpi.com/article/10.3390/biomedicines10071621/s1, Figure S1: (A) Dry weight of partially oxidized alginate hydrogels at different time points. (B) Ramp modulus of partially oxidized alginate hydrogels; Figure S2: Quantification of sGAG and collagen deposition in alginate and oxidized alginate gels following 28 days of in vitro culture; Figure S3: Post printing spreading ratio (width of the filament divided by needle diameter); Figure S4: SEM images at Day 0 and Day 2 revealing the increased porosity after the gelatin removal. The scale bars are equal to 20 μm; Figure S5: Representative images of the live/dead staining at day 7. Green and red indicate live and dead cells, respectively. Scale bars are equal to 250 μm.
