# Supplementary material for: Tuning the Degradation Rate of Alginate-Based Bioinks for Bioprinting Functional Cartilage Tissue
Source: Biomedicines. 2022 Jul 7;10(7):1621. doi: 10.3390/biomedicines10071621 (PMC9312793; doi:10.3390/biomedicines10071621)
Supplement: Supplementary file 1 [file biomedicines-10-01621-s001.zip › biomedicines-1768355-supplementary.pdf]

Supplementary Information:

Tuning the Degradation Rate of Alginate-Based Bioinks for Bioprinting Functional Cartilage Tissue

Xavier Barceló [1,2,3], Kian F. Eichholz [1,2,3], Orquidea Garcia [4] and Daniel J. Kelly [1,2,3,5,*]

[1] Trinity Centre for Biomedical Engineering, Trinity Biomedical Sciences Institute, Trinity College Dublin, D02 R590 Dublin, Ireland; barcelgx@tcd.ie (X.B.); eichholk@tcd.ie (K.F.E.)
[2] Department of Mechanical, Manufacturing & Biomedical Engineering, School of Engineering, Trinity College Dublin, D02 R590 Dublin, Ireland
[3] Advanced Materials & Bioengineering Research Centre (AMBER), Royal College of Surgeons in Ireland & Trinity College Dublin, D02 F6N2 Dublin, Ireland
[4] Johnson & Johnson 3D Printing Innovation & Customer Solutions, Johnson & Johnson Services, Inc., Irvine, CA 92618, USA; ogarci22@its.jnj.com
[5] Department of Anatomy, Royal College of Surgeons in Ireland, D02 YN77 Dublin, Ireland
* Correspondence: kellyd9@tcd.ie; Tel.: +353-18-963-947

Supplementary Figure S1. (A) Dry weight of partially oxidized alginate hydrogels at different time points. (B) Ramp modulus of partially oxidized alginate hydrogels.

Supplementary Figure S2. Quantification of sGAG and collagen deposition in alginate and oxidized alginate gels following 28 days of in vitro culture.

Supplementary Figure S3. Post printing spreading ratio (width of the filament divided by needle diameter).

Supplementary Figure S4. SEM images at Day 0 and Day 2 revealing the increased porosity after the gelatin removal. The scale bars are equal to 20 μm.

Supplementary Figure S5. Representative images of the live/dead staining at day 7. Green and red indicate live and dead cells, respectively. Scale bars are equal to 250 μm.

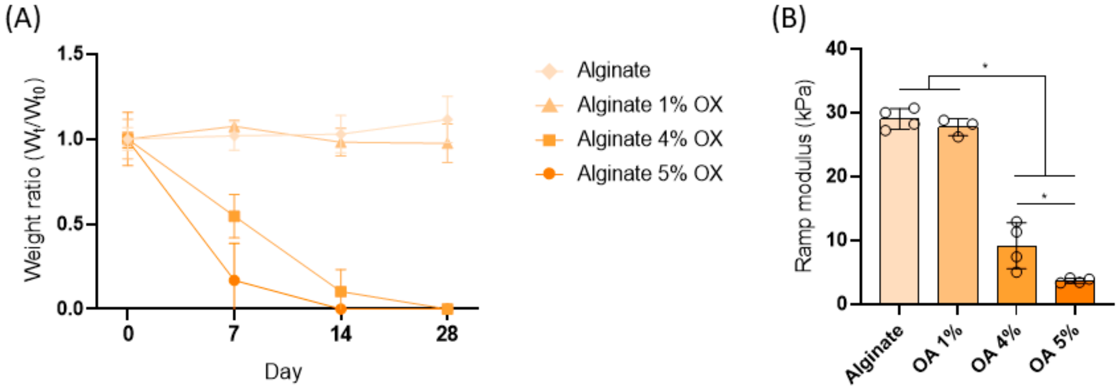

Supplementary Figure S1. (A) Dry weight of partially oxidized alginate hydrogels at different time points. (B) Ramp modulus of partially oxidized alginate hydrogels.

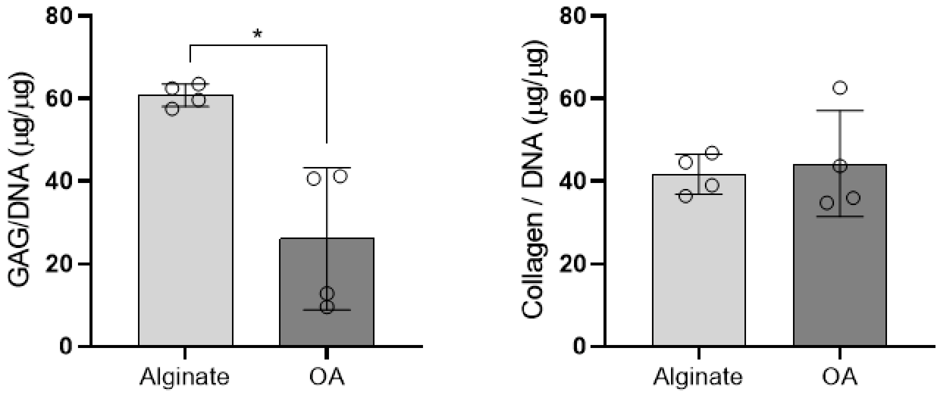

Supplementary Figure S2. Quantification of sGAG and collagen deposition in alginate and oxidized alginate gels following 28 days of in vitro culture.

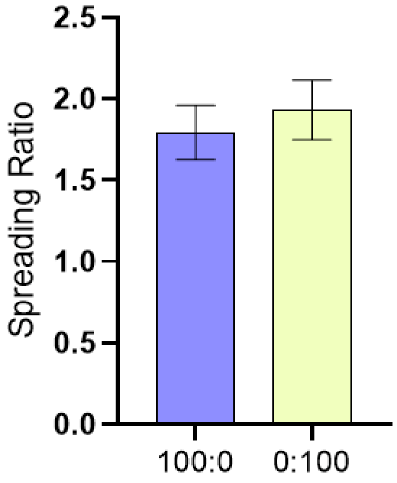

Supplementary Figure S3. Post printing spreading ratio (width of the filament divided by needle diameter).

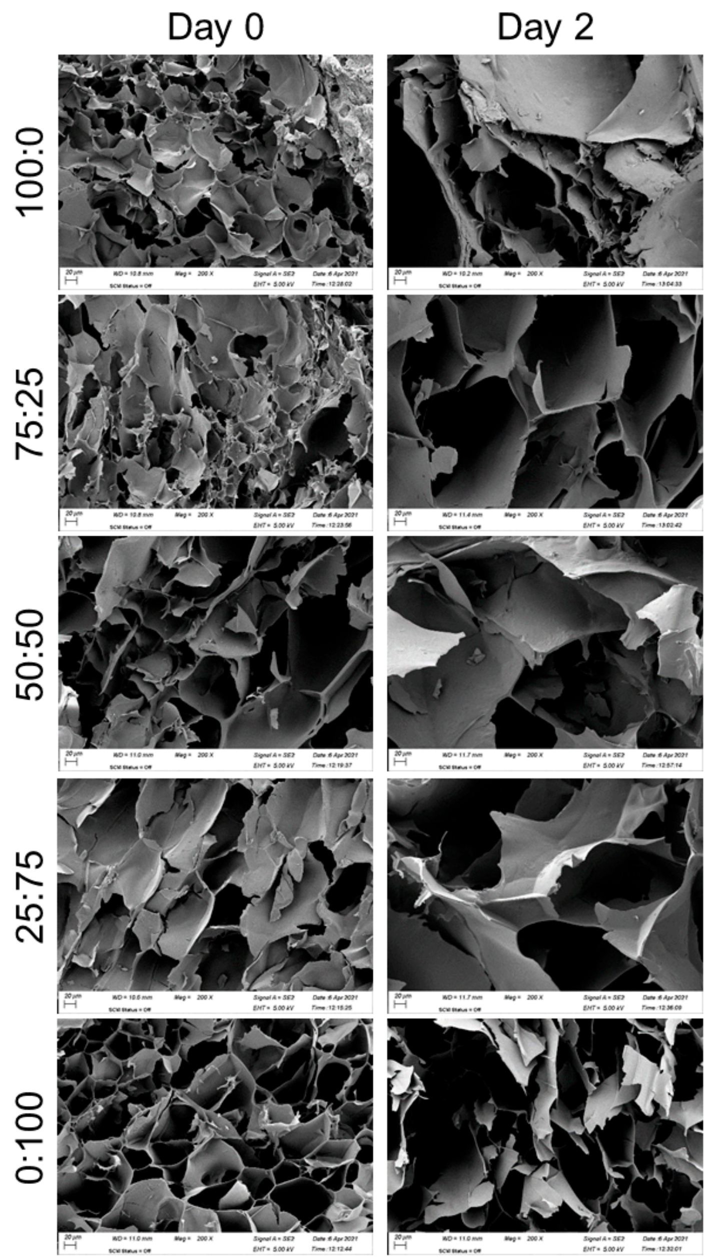

Supplementary Figure S4. SEM images at Day 0 and Day 2 revealing the increased porosity after the gelatin removal. The scale bars are equal to 20 μm.

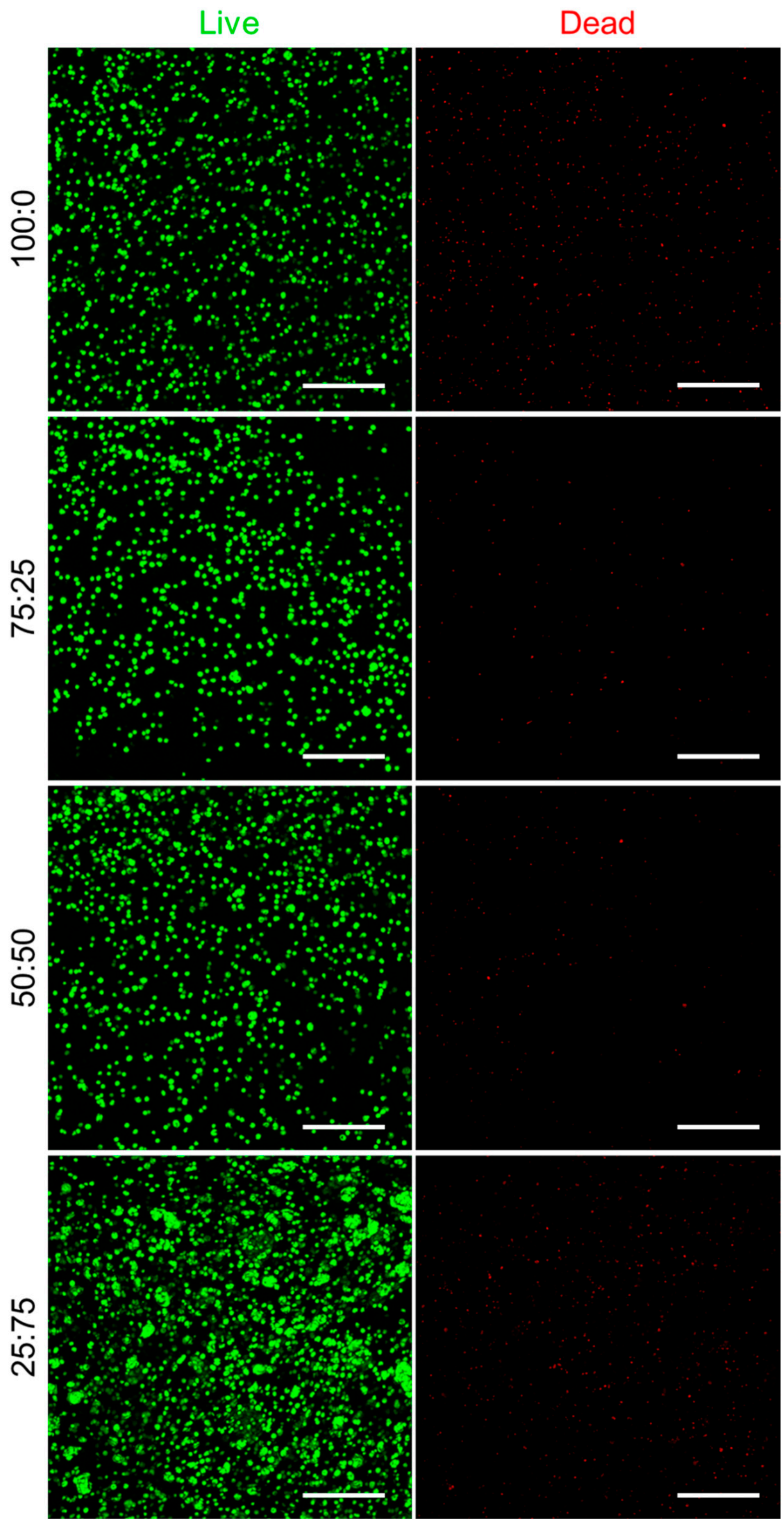

Supplementary Figure S5. Representative images of the live/dead staining at day 7. Green and red indicate live and dead cells, respectively. Scale bars are equal to 250 μm.
